# Eyasi Plateau Paleontological Expedition, Laetoli, Tanzania, fossil specimen database 1998–2005

**DOI:** 10.1038/s41597-019-0304-2

**Published:** 2019-12-03

**Authors:** Denné Reed, Terry Harrison, Amandus Kwekason

**Affiliations:** 1 Department of Anthropology, University of Texas at Austin, Austin, USA; 2 Center for the Study of Human Origins, Department of Anthropology, New York University, New York, USA; 3 National Museum of Tanzania, Dar es Salaam, Tanzania

**Keywords:** Palaeoecology, Palaeoecology, Biological anthropology

## Abstract

The Eyasi Plateau Paleontological Expedition (EPPE) Laetoli specimen database contains 13716 records of plant and animal fossils (ca. 28248 specimens) collected by EPPE field teams working at Laetoli, Tanzania between 1998 and 2005. This dataset is a digital version of the original hard-copy specimen catalog, and it documents the discovery, stratigraphic provenience and taxonomic diversity of Plio-Pleistocene fauna and flora in northern Tanzania between 4.4 Ma and >200 ka. Laetoli is renowned for the discovery of important hominin fossils, including the lectotype for *Australopithecus afarensis*, one of our early hominin ancestors, the first record of *Paranthropus aethiopicus* outside Kenya-Ethiopia, and an early record of our own species *Homo sapiens*. This database is one of the few publicly available palaeoanthropological fossil datasets and serves as an example for expanding open access to primary fossil occurrence data in palaeoanthropology. The taxonomic identifications appearing in this dataset are the original field identifications and are provisional. Any taxonomic analysis employing this dataset should refer to updated taxonomic identifications published by specialists.

## Abstract (summary)

**Table:** 

Measurement(s)	fossil
Technology Type(s)	digital curation
Factor Type(s)	taxonomy • geographic location
Sample Characteristic - Location	Laetoli

Machine-accessible metadata file describing the reported data: 10.6084/m9.figshare.10265336

## Background & Summary

**Fig. 1:**
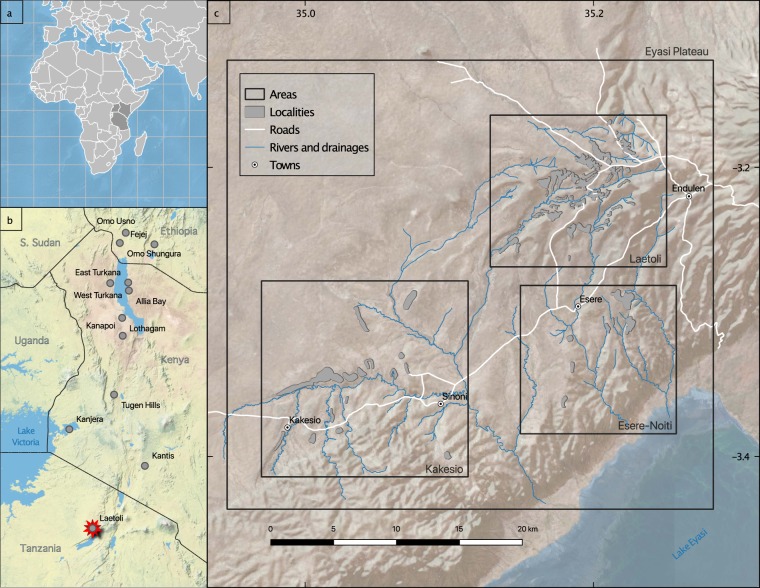
Map showing the location of Laetoli relative to other paleoanthropology fossil sites.

**Fig. 2:**
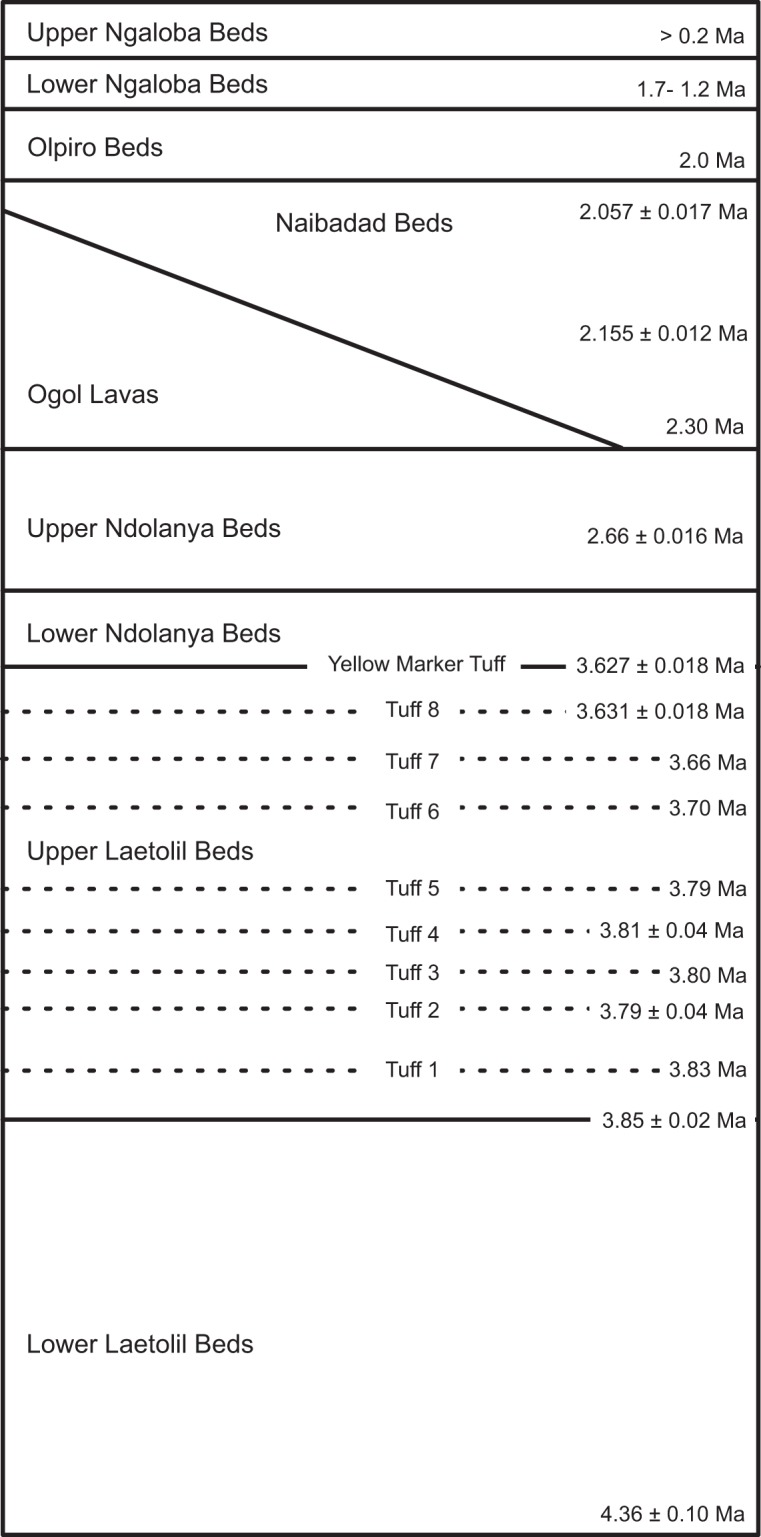
Summary of stratigraphic units and associated radiometric dates at Laetoli. ^40^Ar/^39^Ar dates are given with standard errors.

Laetoli in northern Tanzania is one of the most important palaeontological and palaeoanthropological sites in eastern Africa. The site is located on the Eyasi Plateau, an uplifted fault block at the southern end of the eastern branch of the East African Rift Valley, bordered to the south by Lake Eyasi (Fig. 1). To the east is the Ngorongoro Volcanic Highland Complex, comprising a series of Pliocene and Pleistocene volcanoes. These are the source of the primary air fall tuffs and reworked tuffaceous sediments in which the fossils are buried and preserved^1^ (Fig. 2). Tephra throughout the stratigraphic sequence are amenable to radiometric dating and this has allowed an excellent geochronological framework to be established^2^. Fossils were first discovered in the area in the 1930s (when the site was referred to as Garusi), but the significance of Laetoli for human evolution was not fully appreciated until the late 1970s when Mary Leakey began her field research in the area^3^. The current phase of geological and palaeontological fieldwork, co-directed by TH and AK, was initiated in 1998 and continues to the present-day^4,5^. The database comprises data derived from the first seven years (8 field seasons) of renewed paleontological collections (1998–2005).

Laetoli is perhaps best known for the discovery of important fossil remains and ichnological traces of early hominins. The site has yielded hominins from three stratigraphic units: Upper Laetolil Beds, Upper Ndolanya Beds, and Upper Ngaloba Beds (Fig. 2). The specimens of *Australopithecus afarensis* from the Upper Laetolil Beds (3.85–3.6 Ma) are relatively few, but nevertheless represent one of the largest and geologically oldest assemblages, including the lectotype of the species^6^. The Upper Laetolil Beds also preserve several trails of footprints of *Au. afarensis* that provide direct evidence of early hominin bipedal behaviour^3,7–9^. Hominins from the Upper Ndolanya Beds (2.66 Ma) include the first specimen of *Paranthropus aethiopicus* recovered from outside the Turkana Basin of northern Kenya and Ethiopia, and one of the oldest securely dated specimens attributable to this species^6^. Finally, a partial cranium of an archaic *Homo sapiens* has been recovered from the Late Pleistocene Upper Ngaloba Beds (>200 ka) associated with Middle Stone Age artefacts^10,11^. In addition to the hominins, a rich record of the fossil remains of animals and plants from Laetoli offers important insights into the faunal and floral diversity of Africa during the Pliocene, and provides a well-dated reference for comparisons with other Plio-Pleistocene faunas from Africa and Eurasia.

The database represents an important addition to the resources currently available for researchers investigating human evolution and vertebrate palaeontology in Africa. Field data of this kind, which provide crucial documentation about the nature and history of fossil collections, is rarely available to other researchers, and in the past essential contextual data about historical collections have been lost. For example, in 1938–1939 Ludwig and Margrethe Kohl-Larsen made one of the most important collections of fossil vertebrates from Laetoli^12–14^, which is housed in the Museum für Naturkunde in Berlin and the University of Tübingen. However, no comprehensive documentation of the collecting localities and stratigraphic provenance of the specimens was made at the time of discovery (or at least none that survives to the present-day), so crucial information about the context is largely unknowable and this greatly lessens the value and significance of the Kohl-Larsen collections. Making the Laetoli field data available ensures that future researchers have access to the history and contextual information relating to the discovery of individual specimens. As such, the database becomes an important historical resource. The database also provides important information, especially when combined with corresponding data from other palaeontological sites in Africa, which can be used for analyses of palaeoecology, palaeobiogeography, taphonomy, biochronology, and macroevolutionary patterns of speciation and extinction. Finally, the database can be used in broader-scale analyses of the impact of regional and global climate change on biotas during the Pliocene.

## Methods

**Fig. 3:**
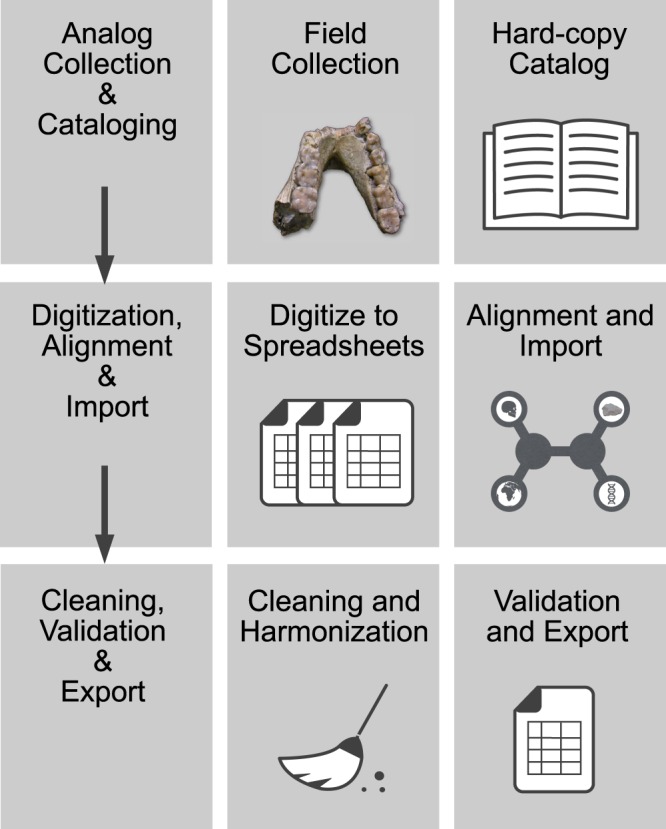
Data collection and processing workflow.

The data were collected and processed following the steps outlined in Fig. 3, from field collection and hard-copy documentation, to digitization, alignment/import, cleaning/harmonization, and metadata annotation.

### Field collection and documentation

The original fossils were recovered from the Eyasi Plateau, an uplifted fault block on the northwest margin of Lake Eyasi, located in the Ngorongoro Conservation Area, Tanzania (3.25°S, 35.10°E). Most of the fossils were recovered from the Laetoli area, but smaller collections have been recovered from Kakesio and Esere-Noiti. The project area covers approximately 400 square km. Fossils were primarily recovered from the surface of exposed outcrops after they have eroded out of the sediments. Partially exposed fossils *in situ* were excavated. No systematic screening for microinvertebrates and microvertebrates was undertaken, although dry screening methods were employed to recover associated remains and at localities where fossil hominins were recovered. The collection protocol stipulates collecting all vertebrate fossils that were anatomically identifiable (with the exception of rib fragments and limb bone shaft fragments that did not retain at least a portion of one articular surface). Bone fragments that were not anatomically identifiable but preserved traces of taphonomic interest (such as carnivore bite marks, rodent gnawing, cut marks, insect damage, and root etching) were also collected. Isolated fragments of tortoise shells and ostrich eggshell, terrestrial gastropods, insects and insect traces, and macrobotanical fossils were not collected systematically, but representative specimens were collected at each locality as reference specimens. Collecting events occurred at 60 designated localities and sublocalities within specific stratigraphic units in those localities^15^. Fossils were cataloged the same day they were discovered and field numbers were inscribed on the fossils with permanent ink. Collection details (date of collection, locality, stratigraphic unit, anatomical element, taxonomic identification, other remarks) for each fossil were written onto collection cards that remain with the fossil and details were also written into a hard-copy collection catalog. Preliminary taxonomic identifications included in the catalog are based on expertise and literature sources. All specimens were accessioned into the collections of the National Museums of Tanzania (NMT), Dar es Salaam.

### Digitization

Original records of the collected materials were written into a hard-copy catalog that is kept at the National Museums of Tanzania (NMT), Dar es Salam, Tanzania. Entries from the paper catalog were digitized into seven spreadsheet files, one for each field campaign in 1998, 1999, 2000, 2001, 2003, 2004, 2005. In all there were eight field campaigns, with two separate campaigns in 2000 (one in January-February and another in August). Data from the spreadsheets were imported into the Paleo Core data repository (http://paleocore.org), aligned to standard fields and harmonized to established vocabularies and formats as described below. In total 13720 records were read from the spreadsheets, of these 10 were deleted as duplicates and 6 records were added as the result of splitting bulk records, resulting in the final count of 13716 data records (Table 1).

**Table 1:** Number of records imported by year.

Year	Record Count (modifications)
1998	1691 (1693 − 2 duplicate)
1999	377
2000	4377 (4374 − 2 duplicates +5 splits)
2001	1576 (1575 + 1 split)
2003	2540
2004	1749 (1754 − 5 duplicates)
2005	1406 (1407 − 1 duplicates)
Total	13716 (13720 − 10 duplicates + 6 splits)

### Alignment and import

Data from the digitized spreadsheets were mapped to a set of verbatim fields that record the original values from the spreadsheets in the Paleo Core database. The data in the verbatim fields were then processed and used to populate the cleaned fields in the database (Table 2). Two (2) of the spreadsheet columns, “Tray” and “Published” contained no data and were dropped.

**Table 2:** Mapping between the 19 columns in the spreadsheet files and the corresponding verbatim fields and cleaned fields in the dataset.

	Spreadsheet Column	Verbatim Field	Cleaned Field
1	Specimen Number	verbatim_specimen_number	catalog_number
2	Date Discovered	verbatim_date_discovered	date_discovered
3	Storage	verbatim_storage	institution_code
4	Tray	N/A	N/A
5	Locality	verbatim_locality	locality
6	Horizon	verbatim_horizon	bed
7	Element	verbatim_element	part_of_organism
8	Kingdom	verbatim_kingdom	kingdom
9	Phylum/Subphylum	verbatim_phylum_subphylum	phylum, subphylum
10	Class	verbatim_class	class
11	Order	verbatim_order	order
12	Family	verbatim_family	family
13	Tribe	verbatim_tribe	tribe
14	Genus	verbatim_genus	genus
15	Species	verbatim_species	specific_epithet
16	Other	verbatim_other	taxon_remarks
17	Comments	verbatim_comments	remarks
18	Published	N/A	N/A
19	Problems	verbatim_problems	problem_remark

Two spreadsheet columns, Tray and Published, contained no data and were dropped.

### Cleaning and harmonization

For each of the 17 columns imported into Paleo Core the data were cleaned, and where appropriate, harmonized to a data encoding scheme and structured vocabulary. Details on this process are described in detail below for each field. Alignment and harmonization were automated in Python, so that every step in the process is documented in code and reproducible from the original Excel files.

## Data Records

### Data files

This dataset comprises a single data file in comma delimited format (.csv). The first row is a header of column names matching standard terms. The dataset is available for download from the Paleo Core data repository at: https://paleocore.org/projects/eppe/ and the figshare data repository^16^ at: 10.6084/m9.figshare.8847935.v2.

### Field definitions

The data fields (columns) included in this dataset are of two types: verbatim fields and cleaned fields. The verbatim fields contain the uncleaned, digitized data copied from the spreadsheets. They provide a record of the digitized version of the paper specimen catalog. The cleaned fields contain data cleaned and harmonized from the verbatim data and mapped to one of the standards listed in Table 3. Details about the processing are described in the sections describing each field, and in the Python code for the import script.

**Table 3:** Standards, namespaces and abbreviations.

Standard	Namespace URI	Abbreviation
Darwin Core	http://rs.tdwg.org/dwc/terms/	dwc
ABCD-EFG	http://www.synthesys.info/ABCDEFG/1.0	abc

All field names are presented in snake_case (i.e. in lower-case and words are joined by underscores) to standardize their presentation and promote readability.

#### Verbatim fields

The spreadsheets contained 19 columns of data. Seventeen (17) columns were copied, unmodified, into corresponding “verbatim” fields in the final dataset (Table 2). Dates in the spreadsheets were a mixture of string values and integer date formats, which were parsed accordingly and converted to a standardized format in the database, all other fields were copied, unmodified, as text. Two (2) of the spreadsheet columns, “Tray” and “Published” contained no data and were dropped.

#### Cleaned fields

In addition to the verbatim fields the dataset includes the fields listed in Online-only Table 1. The data from the verbatim fields were cleaned, validated and used to populate these fields. Unless otherwise indicated, each field is defined according to the Darwin Core standard^17^. One field, verbatim_element, does not conform well to an existing Darwin Core term and was aligned to the term PartOfOrganism from the ABCD-EFG data standard. Two of the taxonomic fields, verbatim_phylum_subphylum and verbatim_tribe also are not represented by Darwin Core terms. The column for verbatim_phylum_subphylum was divided into dwc:phylum and subphylum, the latter is not standard but its definition is similar to that of other taxonomic fields in Darwin Core. Similarly, verbatim_tribe was cleaned and transferred to tribe. The tribe field is particularly significant in the analysis of Plio-Pleistocene bovid faunas where many fossils cannot be identified to genus level but can be identified to ecologically informative tribe level designations. A complete listing of all the cleaned fields and their definitions is provided in Online-only Table 1. Additional comments about each field are provided in the subsequent sections.

#### Catalog number

Field name – catalog_number.

Definition – dwc:catalogNumber.

Catalog Number is the primary key (pk) for the EPPE Laetoli Database and as such is unique for all records, but not guaranteed globally unique outside this dataset. Catalog numbers match the values written on the fossil specimens and take the form shown in Table 4, where EP stands for Eyasi Plateau, <item_number> indicates a three- or four-digit unique (to this dataset) integer, [a-z] indicates an optional, lower-case, lettered part of a specimen (Example 2) and yy indicates a two-digit year. Item numbers less than 100 have leading zeros to the hundreds place, as shown in Example 1, though values may expand to the thousands as indicated in Example 2. There is always a single space between EP and the item number. Leading zeros were retained for consistency with published specimen numbers.

**Table 4:** Formatting and examples of entries in the catalog_number field.

Format	Example 1	Example 2
EP <item_number> [item_part]/<yy>	EP 001/98	EP 1345a/05

#### Institution code

Field name – institution_code.

Definition – dwc:institutionCode.

All values for this field are the string, ‘NMT’ for the National Museums of Tanzania.

#### Collection code

Field name – institution_code.

Definition – dwc:collectionCode.

All values for this field are the string ‘EP’ for Eyasi Plateau. This collection code distinguishes these collections from others made at Laetoli, e.g. earlier Leakey collections under code ‘LAET’ or ‘LIT’.

#### Occurrence remarks

Field name – occurrence_remarks.

Definition – dwc:occurrenceRemarks.

This field is copied from verbatim_comments. No additional processing was applied to the verbatim data.

#### Event date

Field name – event_date.

Definition – dwc:eventDate.

The values for event date are derived from verbatim_date_discovered, which in the spreadsheet files were a mix of dates in number format where the value corresponds to the number of days (or fractions thereof) since a designated start date (which is stored in the workbook), or the entries were string values in the format dd/mm/yy, where dd corresponds to the day, mm to the month and yy to the year, all as two digits. Dates were converted to Python date format using the xlrd Python library depending on the data type of the cell in the spreadsheet (date type vs string type). Each record was validated for a value that fell in the interval between 1998 and 2005 inclusive.

#### Basis of record

Field name – basis_of_record.

Definition – dwc:basisOfRecord.

This is a Darwin Core term used to indicate the type of data record. For this dataset all records have the value ‘FossilSpecimen’, which is from the recommended Darwin Core type vocabulary.

#### Part of organism

Field name – part_of_organism.

Definition – abc:PartOfOrganism.

The part_of_organism field records free text description of the fossil elements preserved for each specimen. As there is no Darwin Core term to handle free text anatomical element descriptions, this field definition is drawn from the Access to Biological Collections Data standard (ABCD) Extended for Geology (EFG). Values in the description field were pared down to 2293 unique descriptions from the 3976 unique entries in verbatim_element. The reduction was accomplished by standardizing or expanding abbreviations for anatomical elements, parts and sides.

#### Organism quantity

Field name – organism_quantity.

Definition – dwc:organismQuantity.

The number of identified fossil specimens (NISP) included with each record. The values in this field derive from the item counts appearing in parentheses in verbatim_element, e.g. “Proximal Ulnae (3)”. The anatomical description is preserved in part_of_organism and the number of specimens appearing in parentheses is recorded in organism_quantity as an integer value, without parentheses.

#### Organism quantity type

Field name – organism_quantity_type.

Definition – dwc:organismQuantityType.

This field is set to ‘NISP’ for all records, indicating the number of identified specimens as the quantity expressed in Organismal Quantity.

#### Country

Field name – country.

Definition – dwc:country.

All values for this field are the string, ‘Tanzania’.

#### Locality

Field name – locality.

Definition – dwc:locality.

A locality is the place within the Laetoli project area from where a specimen was recovered. Harrison and Kweka^15^ list 60 fossil localities at Laetoli, which they group into three major areas: Laetoli, Kakesio, and Noiti-Esere (Fig. 1). Three localities (Ndoroto, Olaltanaudo, Oleisusu) fall outside these areas. The 219 unique entries in verbatim_locality were cleaned to match one of the 65 locality terms in the Laetoli locality vocabulary. The additional 5 entries in the vocabulary correspond to conflated values such as ‘Kakesio 1–6’ which indicate that the fossils came from one of the 6 Kakesio localities but it is unclear which one. Locality names follow one of the formats shown in Table 5.

**Table 5:** Formats and examples for entries in the locality field.

Format	Example 1	Example 2
<place> <number> [number range]	Laetoli 1	Kakesio 2–4
<place> <number> <sublocality>	Laetoli 10 East	Laetoli 13 “Snake Gully”
<place>	Engesha	Garusi Southwest

Locality place and number are always separated by a single space, and ranges are indicated by a single n-dash with no spaces around it. All entries were stripped of leading and trailing whitespace.

#### Bed

Field name – bed.

Definition – dwc:bed.

Each fossil derives from a stratigraphic unit or range of units, which comprise the geological context for the fossil. Most collections were surface finds and their provenience is inferred from the surrounding sediments, adhering matrix, and preservation. Geological samples from volcanic tuffs were submitted for radiometric analysis to determine geochronological age controls for the site^3^. The original 196 unique entries in verbatim_horizon were pared down to 34 unique entries in the cleaned, bed field and the values correspond to the units shown in Fig. 2.

#### Minimum/maximum chronometric age

Field names – minimum_chronometric_age, maximum_chronometric_age.

Definitions – dwc:minimumChronometricAge, dwc:maximumChronometricAge.

The age fields are drawn from the Chronometric Age extension to Darwin Core. The minimum and maximum are based on the absolute age estimates for each unit as illustrated in Fig. 2. The dates provided are the best estimates (central tendency) for the minimum and maximum age respectively. Most ages were determined using ^40^Ar/^39^Ar radiometric dating, with Bayesian interpolation for beds between dated tuffs^2,18^. Dates for the Upper Ngaloba are based on Amino Acid Racemization while dates for the Lower Ngaloba Beds are based on biochronology. Each fossil was ascribed dates based on the bed or interval of beds from which it was recovered.

#### Maximum/maximum chronometric age reference system

Field names – minimum_chronometric_age_reference_system, maximum_chronometric_age_reference_system.

Definitions – dwc:minimumChronometricAgeReferenceSystem, dwc:maximumChronometricAgeReferenceSystem.

All dates for minimum and maximum chronometric age are provided in Ma (megaannum, i.e. millions of years).

#### Chronometric age uncertainty in years

Field name – chronometric_age_uncertainty_in_years.

Definition – dwc:chronometricAgeUncertaintyInYears.

While the max and min ages are provided in Ma, the uncertainty is provided in years to conform with the standard. In cases where uncertainty differed between minimum and maximum ages, or where uncertainty is known only for one of the values, the greatest uncertainty is reported. For example, fossils found in the Laetolil Beds, Upper Unit, Between Tuffs 7 – 8, are bracketed between a maximum age of 3.66 Ma and a minimum age of 3.631 ± 0.018 Ma. The maximum age is interpolated and the minimum age is based on argon-argon analysis and has a standard error of 0.018 Ma. The reported uncertainty for this example is thus 18000 years.

#### Taxonomic fields

Field names – kingdom, phylum, subphylum, class, order, family, tribe, genus, specific_epithet.

Definitions – dwc:kingdom, dwc:phylum, dwc:class, dwc:order, dwc:family, dwc:genus, dwc:specific_epithet.

The taxonomic fields, kingdom, phylum, class etc., record preliminary identifications for each specimen at the designated taxonomic rank and are derived from the **verbatim** taxonomic fields. Spelling and typographical errors were corrected, but no attempt was made to update taxonomic assignments. Values from the verbatim_phylum_subphylum field were split into the cleaned fields phylum and subphylum accordingly. There are no standard terms in Darwin Core or ABCD-EFG for the taxon ranks subphylum and tribe. These ranks are included because of their significance in paleoanthropological faunal analysis. Their definitions are as for the other taxon fields.

#### Scientific name

Field name – scientific_name.

Definition – dwc:scientificName.

This field records full name at the lowest level taxonomic designation available for the specimen. It is derived from the **cleaned** taxonomic fields (unlike the taxonomic fields which derive directly from the verbatim taxonomic fields). For genus and above it provides a single word and for species the complete species name (binomen). An important note about this field is that it does not include designations of uncertainty, for example *Australopithecus* cf. *afarensis* will have scientific_name *Australopithecus afarensis* and identification_qualifier cf. *afarensis*, as per the recommended best practice under Darwin Core. Users are encouraged to consult the Darwin Core documentation for dwc:scientificName and dwc:identificationQualifier for definitions and details.

#### Identification qualifier

Field name – identification_qualifier.

Definition – dwc:identificationQualifier.

This field is derived from the verbatim taxonomic fields. The verbatim taxonomic fields were searched for incidents of ‘?, cf., aff, indet., sp., nov.’ and where found the taxonomic field and identification_qualifier field were updated accordingly. The import script includes detailed notes on the regular expression searches and parsing used to update this field. Generally, the identification qualifiers are taken to follow common convention for open nomenclatures *sensu* Sigovini *et al*.^19^. Users are referred to the Darwin Core documentation on dwc:scientificName and dwc:identificationQualifier to understand their definition and use.

#### Taxon rank

Field name – taxon_rank.

Definition – dwc:taxonRank.

This field was updated by analysing the taxon fields to identify the most precise rank for which there is a value. All ranks are presented in lower case.

#### Taxon remarks

Field name – taxon_remarks.

Definition – dwc:taxonRemarks.

The values in this field were updated for cases where taxonomic changes were made such that taxonomic fields differ from verbatim taxon fields.

#### Problem remarks

Field name – problem_remarks.

Definition – Remarks describing potential or known problems with a record.

This field derives from verbatim_problems and provide free text remarks about possible or known problems associated with a data record. Twenty records have entries for verbatim_problems, two of these referring to duplicate catalog numbers were removed. The remaining 18 problem remarks indicate potential missing or problematic fossil specimens. There is no standard term in Darwin Core or ABCD-EFG that matches this field. It could be accommodated in remarks, but we preferred to keep problem remarks separate to facilitate search.

## Technical Validation

Automated validation was conducted for catalog_number, event_date, locality, and the taxonomic fields.

### Catalog number

All catalog_number strings were validated for consistency of formatting and uniqueness.

Entries in the catalog_number field were copied from verbatim_specimen_number and validated against the following Python regular expression:

EP \d{3,4}[a-zA-Z]?/[09][01234589]$

Leading zeros were retained for consistency with published specimen numbers.

#### Splits

Five items for bulk collections contained multiple or mixed taxa and had to be split into parts. The splits added 6 new items to the catalog (Table 6).

**Table 6:** Specimens that were split into new records. Five (5) items were split resulting in 6 new records.

	Catalog Number	Locality	Description	Taxon	Updated to
1	EP 1280/01	Laetoli 9 South	shells (2)	Achatina zanzibarica	1280a/01
			shells (5)	Pseudoglessula cf. gibbonsi	1280b/01
2	EP 3129/00	Laetoli 10	shells (8)	Achatina zanzibarica	EP 3129a/00
			shell (1)	Pseudoglessula cf. gibbonsi	EP 3129b/00
3	EP 1181/00	Laetoli 8	shell (1)	Achatina zanzibarica	EP 1181a/00
				Burtoa nilotica	EP 1181b/00
4	EP 3635/00	Kakesio 5	shell (1)	Achatina zanzibarica	EP 3635a/00
			shels (8)	Limicolaria martensiana	EP 3635b/00
5	EP 1177/00	Laetoli 8	nuchal bone	Stigmochelys brachygularis	EP 1177a/00
			xiphiplastron	Stigmochelys brachygularis	EP 1177b/00
			fragment of carapace	Stigmochelys brachygularis	EP 1177c/00

#### Corrections

Six (6) specimens raised validation errors (Table 7). Three (3) of these specimens had an entry in catalog_number that did not match the formatting regular expression. The first, with verbatim specimen number EP 120A + B/98 is an associated set of left and right upper teeth and skull fragments. These were published^20^ as parts A and B but exact designation for all elements was not given so the catalog number is merged in the official version of the catalog to simply EP 120/98. The other formatting errors are typos in the year suffix, which were corrected. Another three (3) were duplicate catalog numbers resulting from digitization errors. These were corrected as shown in Table 7.

**Table 7:** Corrected catalog numbers.

	Catalog Number	Locality	Description	Taxon	Updated to
1	EP 120A + B/98	Laetoli 10 East	assoc. teeth and cranial fragments	Rhinocerotidae	EP 120/98 and comment added.
2	EP 507/07	Laetoli 9 South	scute	Chelonia	EP 507/05
3	EP 756/06	Laetoli 5	lower incisor	Madoqua	EP 756/05
4	EP 1075/03	Laetoli 10 West	proximal phalanx	Rodentia	same
	EP 1075/03	Laetoli 7	distal metapodials	Serengetilagus	EP 1975/03
5	EP 348/04	Laetoli 8	proximal ulnae	Serengetilagus	same
	EP 348/04	Laetoli 8	distal radius	Serengetilagus	EP 349/04
6	EP 2188/99	Laetoli 7	distal humerus	Bovidae	EP 2188/03
	EP 2188/99	Laetoli 7 East	vertebral centrum	Bovidae	EP 2188/00

#### Deletions

Ten (10) records were deleted from the dataset. Seven (7) records were duplicate entries with identical data (Table 8).

**Table 8:** Deleted duplicate records, one copy of each was deleted.

	Duplicate Catalog Numbers (duplicate deleted)
1	EP 1582b/00
2	EP 1144/04
3	EP 1173/04
4	EP 1400/04
5	EP 1403/04
6	EP 1542/04
7	EP 515/05

Two (2) pairs of duplicate records were the result of emended taxonomic identifications (Table 9, items 1–2). The amended data was retained, the earlier version deleted, and a note of the change was added to the taxonomic remarks field. One (1) duplicate stems from an incorrect entry, which was deleted (Table 9, item 3).

**Table 9:** Deleted and corrected duplicate records reflecting amended taxonomic identifications.

	Catalog Number	Locality	Description	Taxon	Updated to
1	EP 001/98	Kakesio 1	vertebra	Crocodylidae	deleted
	EP 001/98	Kakesio 1	cervical vertebra	Rhinocerotidae	retained with comments added
2	EP 1477b/00	Laetoli 7 East	metatarsal	Suidae	deleted
	EP 1477b/00	Laetoli 7 East	right metatarsal iii	Felidae	retained with comments added
3	EP 1052/98	Laetoli 9 South	calcanei	Serengetilagus	—
	EP 1052/98	Laetoli 9 South	middle phalanx	Aves	deleted

### Date discovered

Discovery dates were automatically validated for formatting and appropriate time intervals, i.e. all dates were tested to fall in the range between 1 Jan 1998 and 31 Dec 2005. Dates were converted to python date objects in the Paleo Core database, and exported as text strings in ISO 8601:2004(E) format.

### Locality

Locality values were validated against the Laetoli locality vocabulary listed in Online-only Table 2. The Verbatim Locality column indicates the corresponding entries in the verbatim_locality field that were harmonized to a common locality name. For example ‘Kakesio #1’ and ‘Kakesio #1’ were both harmonized to the value of ‘Kakesio 1’. Some verbatim entries are identical except for trailing whitespaces.

### Taxonomic fields

Entries in the taxonomic fields were validated for spelling and taxonomic placement (in the appropriate hierarchy) against the iDigBio taxonomic backbone at genus level and above. Taxonomic validation was used to verify taxa, their spellings and their appropriate ranks. Three genera were not found in the iDigBio taxonomic backbone; these were confirmed against the original print catalog and retained.

## Usage Notes

These data represent the primary digital version of the specimen catalog and are suitable for analyses on differences between localities and comparisons with other fossil catalogs from Laetoli and other sites.

The taxonomic identifications and item descriptions are provisional and should be used with care. Taxonomic identifications are provided primarily to assist in data discovery and to assist taxonomic specialists and systematists in identifying specimens in their area of expertise or that might be relevant for subsequent analysis. Quantitative analyses making use of taxonomic information at higher levels (e.g. Tribe and above) may be appropriate but lower-level, fine-grained taxonomic analysis using these data is probably not appropriate without consulting published analyses of the fossils conducted by specialists^5^.

Similarly, the descriptions of anatomical elements preserved is meant as a guide to data discovery and further analysis. Quantitative analysis of element preservation or taphonomic processes based on these data is not recommended without further reference to published analyses of the specimens or confirmation with the physical specimens.

The digital version of this dataset is public and distributed under a Creative Commons CC-0 license^16^. The physical fossils specimens are under the jurisdiction of the National Museums of Tanzania and the Antiquities Division of the Ministry of Natural Resources and Tourism.

## Data Availability

Data were imported into the Paleo Core data repository using Python (version 3.6) standard libraries (re, datetime, pytz), the xlrd library (version 1.20) for reading Excel files, and the database API included with the Django web framework (version 1.11.20). All of the original source code used to process the data are freely and publicly available through the Paleo Core github repository at: https://github.com/paleocore/paleocore110/blob/master/eppe/import_1998_2005.py.

## Online-only Tables

**Online-only Table 1:** Cleaned fields and their definitions.The cleaned fields are named and defined according to existing standards.

	Field Name	Standard Term
1	catalog_number	dwc:catalogNumber
2	institution_code	dwc:institutionCode
3	collection_code	dwc:collectionCode
4	occurrence_remarks	dwc:occurrenceRemarks
5	event_date	dwc:eventDate
6	basis_of_record	dwc:basisOfRecord
7	part_of_organism	abc:PartOfOrganism
8	organism_quantity	dwc:organismQuantity
9	organism_quantity_type	dwc:organismQuantityType
10	country	dwc:country
11	locality	dwc:locality
12	bed	dwc:bed
13	minimum_chronometric_age	dwc:minimumChronometricAge
14	minimum_chronomietric_age_reference_system	dwc:minimumChronometricAgeReferenceSystem
15	maximum_chronometric_age	dwc:maximumChronometricAge
16	maximum_chronometric_age_reference_system	dwc:maximumChronometricAgeReferenceSystem
17	chronometric_age_uncertainty_in_years	dwc:chronometricAgeUncertaintyInYears
18	kingdom	dwc:kingdom
19	phylum	dwc:phylum
20	subphylum	See comments for definition
21	class	dwc:class
22	order	dwc:order
23	family	dwc:family
24	tribe	See comments for definition
25	genus	dwc:genus
26	specific_epithet	dwc:specificEpithet
27	scientific_name	dwc:scientificName
28	identification_qualifier	dwc:identificationQualifier
29	taxon_rank	dwc:taxonRank
30	taxon_remarks	dwc:taxonRemarks
31	problem_remarks	See comments for definition

The abbreviations prefixing each term indicate the standard and its namespace. For these terms, the URIs also serve as URLs, so definitions can be found by following the links for each term, e.g. the definition for catalog_number is available at dwc:catalogNumber = http://rs.tdwg.org/dwc/terms/catalogNumber. The field names are presented in snake_case while the standard terms are presented in their original format (e.g. camelCase).

**Online-only Table 2:** Laetoli locality vocabulary listing all unique values for locality in the database and their mapping to the verbatim_locality entries.

	Locality Name	Area	Verbatim Locality
1	Emboremony 1	Kakesio	‘Emboremony 1’
2	Emboremony 2	Kakesio	‘Emboremony 2’
3	Emboremony 3	Kakesio	‘Emboremony 3’
4	Engesha	Esere-Noiti	‘Engesha’
5	Esere 1	Esere-Noiti	‘Esere 1’; ‘Esere’
6	Esere 2	Esere-Noiti	‘Esere 2’
7	Esere 3	Esere-Noiti	‘Esere 3’
8	Garusi Southwest	Laetoli	‘Garusi R., Sw Of Norsigidok’
9	Kakesio 1	Kakesio	‘Kakesio #1’; ‘Kakesio # 1’
10	Kakesio 10	Kakesio	‘Kakesio #10’; ‘Kakesio #10’; ‘Kakesio 10’; ‘Kakesio #10’
11	Kakesio 1–6	Kakesio	‘Kakesio 1–6’; ‘Kakesio #1–6’; ‘Kakesio 1–6’
12	Kakesio 2	Kakesio	‘Kakesio # 2’; ‘Kakesio #2’; ‘Kakesio 2’
13	Kakesio 2–4	Kakesio	‘Kakesio 2–4’
14	Kakesio 3	Kakesio	‘Kakesio #3’; ‘Kakesio # 3’
15	Kakesio 4	Kakesio	‘Kakesio # 4’
16	Kakesio 5	Kakesio	‘Kakesio 5’; ‘Kakesio #5’
17	Kakesio 6	Kakesio	‘Kakesio #6’; ‘Kakesio 6’
18	Kakesio 7	Kakesio	‘Kakesio 7’
19	Kakesio 8	Kakesio	‘Kakesio 8’; ‘Kakesio #8’; ‘Kakesio 8’
20	Kakesio 9	Kakesio	‘Kakesio 9’
21	Kakesio South	Kakesio	‘Kakesio South’
22	Laetoli 1	Laetoli	‘Laetoli Loc. 1’; ‘Laetoli Loc. 1’; ‘Loc. 1’; ‘Laetolil Loc 1’
23	Laetoli 10	Laetoli	‘Laetolil Loc. 10’; ‘Laetoli Loc. 10’; ‘Loc. 10’
24	Laetoli 10 East	Laetoli	‘Laetoli Loc. 10E’; ‘Laetoli Loc. 10 E’; ‘Laetoli Loc. 10 E’; ‘Loc. 10E’; ‘Loc. 10E’; ‘Laetoli Loc. 10E’
25	Laetoli 10 Northeast	Laetoli	‘Laetoli Loc. 10 Ne’
26	Laetoli 10 West	Laetoli	‘Laetoli Loc. 10W’; ‘Laetolil Loc. 10W’; ‘Laetoli Loc. 10W’; ‘Laetoli Loc. 10W’; ‘Laetoli Loc. 10W’; ‘Loc. 10W’; ‘Laetolil Loc. 10W’
27	Laetoli 11	Laetoli	‘Laetoli Loc 11’; ‘Laetoli Loc. 11’; ‘Laetoli Loc. 11’; ‘Laetoli Loc 11’; ‘Loc. 11’; ‘Laetoli Loc 11’
28	Laetoli 12	Laetoli	‘Laetoli Loc. 12’; ‘Laetoli Loc. 12’; ‘Laetoli Loc. 12’; ‘Laetoli Loc. 12’; ‘Loc. 12’
29	Laetoli 12 East	Laetoli	‘Laetoli Loc. 12E’; ‘Laetoli Loc. 12 E’; ‘Laetoli Loc. 12E’; ‘Loc. 12E’
30	Laetoli 13	Laetoli	‘Laetoli Loc. 13’; ‘Loc. 13E’; ‘Laetoli Loc. 13’; ‘Laetoli Loc. 13/14’; ‘Laetoli Loc. 13’; ‘Laetoli Loc. 13E’; ‘Laetoli Loc. 13 E’; ‘Loc. 13’; ‘Laetoli Loc. 13’
31	Laetoli 13 “Snake Gully”	Laetoli	‘Laetoli Loc. 13, “Snake Gully”; ‘Laetoli Loc. 13’, “Snake Gulley”; ‘Loc. 13’, “snake gully”
32	Laetoli 14	Laetoli	‘Laetoli Loc. 14’; ‘Laetoli Loc. 14’; ‘Loc. 14’; ‘Laetoli Loc. 14’
33	Laetoli 15	Laetoli	‘Loc. 15’; ‘Laetoli Loc. 15’; ‘Laetoli Loc. 15’; ‘Loc. 15’; ‘Laetoli Loc. 15’; ‘Laetoli Loc. 15’; ‘Laetoli Loc. 15’
34	Laetoli 16	Laetoli	‘Laetoli Loc. 16’; ‘Laetoli Loc 16’; ‘Laetoli Loc 16’; ‘Laetoli Loc. 16’; ‘Loc. 16’; ‘Laetoli Loc. 16’; ‘Loc 16’
35	Laetoli 17	Laetoli	‘Loc. 17’; ‘Laetoli Loc 17’; ‘Laetoli Loc. 17’; ‘Laetoli Loc. 17’
36	Laetoli 18	Laetoli	‘Loc. 18’; ‘Loc. 18’; ‘Laetoli Loc. 18, Gadjingero Gully’; ‘Loc. 18, east side’; ‘Laetoli Loc. 18, Upper Flank’; ‘Loc. 18’; ‘Laetoli Loc. 18’; ‘Laetoli Loc. 18’; ‘Laetoli Loc. 18, Northern Flank’
37	Laetoli 19	Laetoli	‘Loc. 19’; ‘Laetoli Loc. 19’; ‘Laetoli Loc. 19’; ‘Laetoli Loc. 19’
38	Laetoli 1 Northwest	Laetoli	‘Loc. 1NW’
39	Laetoli 2	Laetoli	‘Laetoli Loc. 2’; ‘Laetoli Loc. 2, Nw Gully (=Loc. 2W)’; ‘Laetoli Loc. 2 (West)’; ‘Laetoli Loc. 2, South Gully (=Loc. 2S)’; ‘Loc. 2 (S)’; ‘Laetoli Loc. 2 (?West)’; ‘Laetoli Loc. 2 (West)’; ‘Laetoli Loc. 2’; ‘Laetoli Loc. 2 (South)’; ‘Loc. 2 (NW)’
40	Laetoli 20	Laetoli	‘Laetoli Loc. 20’; ‘Laetoli Loc.20’
41	Laetoli 21	Laetoli	‘Loc. 21’; ‘Laetoli Loc.21’; ‘Laetoli Loc. 21’; ‘Laetoli Loc.21’; ‘Laetoli Loc. 21’
42	Laetoli 21 And 21 East	Laetoli	‘Laetoli Loc. 21 And 21E’
43	Laetoli 22	Laetoli	‘Laetoli Loc. 22’; ‘Loc. 22’; ‘Laetoli Loc. 22’; ‘Laetoli Loc. 22’; ‘Laetoli Loc. 22’; ‘Laetoli Loc. 22’
44	Laetoli 22 East	Laetoli	‘Laetoli Loc. 22E’; ‘Laetoli Loc. 22 E’; ‘Laetoli Loc. 22 E’; ‘Laetoli Loc. 22 E’; ‘Laetoli Loc. 22E’
45	Laetoli 22 South	Laetoli	‘Loc. 22S’; ‘Laetoli Loc. 22S’; ‘Laetoli Loc. 22S’; ‘Laetoli Loc. 22S’; ‘Laetoli Loc. 22S’
46	Laetoli 22 South Nenguruk Hill	Laetoli	‘Laetoli Loc. 22S, Nenguruk Hill’; ‘Loc. 22 South (Nenguruk Hill)’
47	Laetoli 23	Laetoli	‘Laetoli Loc. 23’; ‘Loc. 23’; ‘Laetoli Loc. 23’
48	Laetoli 24	Laetoli	‘Laetoli Loc. 24’
49	Laetoli 3	Laetoli	‘Loc. 3’; ‘Laetolil Loc. 3’; ‘Laetoli Loc. 3’; ‘Laetoli Loc. 3’
50	Laetoli 4	Laetoli	‘Laetoli Loc 4’; ‘Loc. 4’; ‘Loc. 4’; ‘Laetoli Loc. 4’; ‘Laetoli Loc. 4’
51	Laetoli 5	Laetoli	‘Laetoli Loc. 5’; ‘Laetoli Loc. 5’; ‘Laetoli Loc 5’; ‘Laetoli Loc. 5’; ‘Laetoli Loc. 5’; ‘Loc. 5’; ‘Gulley 0.5 km north of Loc. 5’
52	Laetoli 6	Laetoli	‘Loc. 6’; ‘Laetoli Loc. 6’; ‘Laetoli Loc. 6’; ‘Laetoli Loc. 6’; ‘Laetoli Loc. 6’
53	Laetoli 7	Laetoli	‘Laetoli Loc. 7 Upper Laetolil Beds between Tuffs 5 + 7’; ‘Laetolil Loc. 7’; ‘Laetoli Loc. 7’; ‘Loc. 7’; ‘Laetoli Loc. 7’
54	Laetoli 7 East	Laetoli	‘Laetoli Loc. 7E’; ‘Laetolil Loc. 7E’; ‘Loc. 7E’; ‘Laetoli Loc. 7 E’
55	Laetoli 8	Laetoli	‘Laetoli Loc 8’; ‘Loc. 8’; ‘Loc. 8’; ‘Laetoli Loc 8’; ‘Laetoli Loc. 8’
56	Laetoli 9	Laetoli	‘Laetoli Loc. 9’; ‘Laetoli Loc 9’; ‘Laetoli Loc.9’; ‘Laetoli Loc.9’; ‘Laetoli Loc. 9’; ‘Loc. 9’
57	Laetoli 9 South	Laetoli	‘Laetoli Loc. 9’; ‘Laetoli Loc.9’; ‘Laetoli Loc. 9 S’; ‘Laetoli Loc. 9S’; ‘Laetoli Loc. 9’; ‘Upper Ngaloba Beds? (Th)’; ‘Laetoli 9S’; ‘Laetoli Loc. 9S’; ‘Loc. 9S’; ‘Laetoli Loc.95’
58	Lobileita	Kakesio	‘Lobileita’; ‘Lobeleita’
59	Ndoroto	Ndoroto	‘Ndoroto’
60	Noiti 1	Esere-Noiti	‘Noiti’
61	Noiti 3	Esere-Noiti	‘Noiti 3’
62	Olaitole River Gully	Laetoli	‘Olaitole River Gully’
63	Olaltanaudo	Olaltanaudo	‘Olaltanaudo’
64	Oleisusu	Oleisusu	‘Oleisusu’; ‘Olesuisu’
65	Silal Artum	Laetoli	‘Silal Artum’; ‘Silal Artum’

The contents of this table were generated by running validate_locality(verbose = True) from the import script.

## References

[1] Ditchfield, P. & Harrison, T. Sedimentology, Lithostratigraphy and Depositional History of the Laetoli Area. In *Paleontology and Geology of Laetoli: Human Evolution in Context: Volume 1: Geology, Geochronology, Paleoecology and Paleoenvironment* (ed. Harrison, T.) 47–76 (Springer Netherlands, 2011).

[2] Deino, A. L. ^40^Ar/^39^Ar Dating of Laetoli, Tanzania. In *Paleontology and Geology of Laetoli: Human Evolution in Context: Volume 1: Geology, Geochronology, Paleoecology and Paleoenvironment* (ed. Harrison, T.) 77–97 (Springer Netherlands, 2011).

[3] Leakey, M. D. & Harris, J. M. *Laetoli, a Pliocene site in northern Tanzania* (1987).

[4] Harrison, T. (Ed). *Paleontology and Geology of Laetoli: Human Evolution in Context: Volume 1: Geology, Geochronology, Paleoecology and Paleoenvironment*. (Springer, Dordrecht, 2011).

[5] Harrison, T. (Ed). *Paleontology and Geology of Laetoli: Human Evolution in Context: Volume 2: Fossil Hominins and the Associated Fauna*. (Springer, Dordrecht, 2011).

[6] Harrison, T. Hominins from the Upper Laetolil and Upper Ndolanya Beds, Laetoli. In *Paleontology and Geology of Laetoli: Human Evolution in Context: Volume 2: Fossil Hominins and the Associated Fauna* (ed. Harrison, T.) 141–188 (Springer Netherlands, 2011).

[7] Leakey MD, Hay RL (1979). Pliocene footprints in the Laetolil Beds at Laetoli, northern Tanzania. Nature.

[8] Hay, R. & Leakey, M. The fossil footprints of Laetoli. *Sci. Am*. **246**, 50–57 (1982).

[9] Masao, F. T. *et al*. New footprints from Laetoli (Tanzania) provide evidence for marked body size variation in early hominins. *Elife* **5**, 1–29 (2016). 10.7554/eLife.19568 PMC5156529 27964778

[10] Day MH, Leakey MD, Magori C (1980). A new hominid fossil skull (LH 18) from the Ngaloba Beds, Laetoli, northern Tanzania. Nature.

[11] Magori CC, Day MH (1983). Laetoli Hominid 18: an early Homo sapoens skull. J. Hum. Evol..

[12] Dietrich, W. O. Die säugetierpaläontologischen Ergebnisse der Kohl-Larsen’schen Expedition 1937–1939 im nördlichen Deutsch-Ostafrika. *Neues Jahrb. Mineral. Geol. Palaeontol. Abh. Abt. B*, **8**, 217–223 (1941).

[13] Dietrich WO (1942). Ältestquartäre Säugetiere aus der südlichen Serengeti, Deutsch-Ostafrika. Palaeontographica.

[14] Kohl-Larson, L. *Auf den Spuren des Vormenschen Forshungen, Fahrten und Erlebnisse in Deutsch-Ostafrika*. (Strecker und Schröder, 1943).

[15] Harrison, T. & Kweka, A. Paleontological Localities on the Eyasi Plateau, Including Laetoli. In *Paleontology and Geology of Laetoli: Human Evolution in Context: Volume 1: Geology, Geochronology, Paleoecology and Paleoenvironment* (ed. Harrison, T.) 17–45 (Springer Netherlands, 2011).

[16] Reed D, Harrison T, Kwekason A (2019). figshare.

[17] Wieczorek J (2012). Darwin Core: An Evolving Community-Developed Biodiversity Data Standard. PLoS One.

[18] Mollel, G. F., Swisher, C. C., Feigenson, M. D. & Carr, M. J. Petrology, Geochemistry and Age of Satiman, Lemagurut and Oldeani: Sources of the Volcanic Deposits of the Laetoli Area. In *Paleontology and Geology of Laetoli: Human Evolution in Context: Volume 1: Geology, Geochronology, Paleoecology and Paleoenvironment* (ed. Harrison, T.) 99–119 (Springer Netherlands, 2011).

[19] Sigovini M, Keppel E, Tagliapietra D (2016). Open Nomenclature in the biodiversity era. Methods Ecol. Evol..

[20] Hernesniemi, E., Giaourtsakis, I. X., Evans, A. R. & Fortelius, M. Rhinocerotidae. In *Paleontology and Geology of Laetoli: Human Evolution in Context: Volume 2: Fossil Hominins and the Associated Fauna* (ed. Harrison, T.) 275–294 (Springer Netherlands, 2011).

